# Role of Bending Energy and Knot Chirality in Knot Distribution and Their Effective Interaction along Stretched Semiflexible Polymers

**DOI:** 10.3390/polym8100347

**Published:** 2016-09-22

**Authors:** Saeed Najafi, Rudolf Podgornik, Raffaello Potestio, Luca Tubiana

**Affiliations:** 1 Max Planck Institute for Polymer Research, Ackermannweg 10, 55128 Mainz, Germany; najafi@mpip-mainz.mpg.de (S.N.); potestio@mpip-mainz.mpg.de (R.P.); 2 Department of Physics, Faculty for Mathematics and Physics, University of Ljubljana, SI-1000 Ljubljana, Slovenia; rudolf.podgornik@fmf.uni-lj.si; 3 Department of Theoretical Physics, J. Stefan Institute, SI-1000 Ljubljana, Slovenia; 4 Computational Physics Group, University of Vienna, Sensengasse 8, 1090 Vienna, Austria

**Keywords:** semiflexible polymers, dsDNA, knots, chirality, topology

## Abstract

Knots appear frequently in semiflexible (bio)polymers, including double-stranded DNA, and their presence can affect the polymer’s physical and functional properties. In particular, it is possible and indeed often the case that multiple knots appear on a single chain, with effects which have only come under scrutiny in the last few years. In this manuscript, we study the interaction of two knots on a stretched semiflexible polymer, expanding some recent results on the topic. Specifically, we consider an idealization of a typical optical tweezers experiment and show how the bending rigidity of the chain—And consequently its persistence length—Influences the distribution of the entanglements; possibly more importantly, we observe and report how the relative chirality of the otherwise identical knots substantially modifies their interaction. We analyze the free energy of the chain and extract the effective interactions between embedded knots, rationalizing some of their pertinent features by means of simple effective models. We believe the salient aspect of the knot–knot interactions emerging from our study will be present in a large number of semiflexible polymers under tension, with important consequences for the characterization and manipulation of these systems—Be they artificial or biologica in origin—And for their technological application.

## 1. Introduction

A general comprehension of the statistical behavior of semiflexible polymers strongly stretched by an externally imposed tension is relevant to understanding the details of DNA processing in cells [1], as well as to quantify their behavior in single molecule experiments [2]. In the latter, DNA is standardly prepared in a linear, topologically trivial form; on the contrary, the vagaries of the intracellular [3,4,5,6] and viral DNA environment [7,8,9,10] suggest that we also need to focus our attention on more complicated, knotted forms of DNA [6,11,12]. In this context, the behavior of single isolated knots on DNA has received plenty of attention [13,14]; in particular regarding the size, distribution, and dynamics of different prime knots [15,16,17,18,19,20,21,22,23,24,25,26], while more probable [27] and experimentally observed [28] composite knots—Topological entanglements made of multiple prime knots—Have remained much less scrutinized.

When multiple knots are present on a finite-size polymer, they do not behave like independent point-like decorations, as theorized for infinitely long polymer rings [29,30]. Instead, they show a propensity to either intertwine or repel each other, which is controlled by the polymer size [31], its bending energy [32] and its electrostatic self-repulsion [33]. These features suggest that finite size effects are relevant in most biological phenomena and nanotechnological applications involving stiff polymers such as double-stranded DNA (dsDNA), and that indeed the overall effect of the presence of knots on the chain may depend on some complex interaction between them.

The questions we will address in what follows are connected with the details of the free energy pertaining to composite knots tied on a semiflexible polymer, which was shown to exhibit a minimum for intertwined knots [32]. Specifically, we will be concerned with the nature of the effective interaction between prime components of the composite knots that was recently discovered to exhibit unexpected features: apart from depending on the bending stiffness of the underlying polymer, it was shown to depend also on the *relative chirality* of the interacting knots [34]. In fact, in our recent work [34], we found that not only do two knots on a stretched semiflexible polymer under external tension present a free energy minimum when intertwined, but, more significantly, that the depth of this minimum depends on the relative chirality of the two knots. This minimum is more pronounced for knots of opposite chiralities, and the chirality-dependent features of the interaction are present even for otherwise identical trefoil knots.

The chirality effect originates from a complicated dependence of the bending energy on the configuration of the interacting knots, which, within the intertwined knots, is arguably caused by a different arrangement of the inner (nested) knot. As a corollary, this finding implies that torus knots with opposite chiralities should remain intertwined longer than knots with the same chirality—A fundamental feature of the interaction between knots that should change our perspective on their overall importance and salient features.

In order to characterize the role of chirality and chain stiffness on the properties of the composite knots, we simulate a typical optical tweezers experiment involving a knotted polymer [16,26,32]. In particular, we consider two different topologies: A $3_{1}^{+}3_{1}^{+}$ and a $3_{1}^{+}3_{1}^{-}$ knot, distinguished only by the relative chiralities of their components, for different degrees of polymer stiffness. By means of molecular dynamics (MD) simulations in implicit solvent with an underdamped Langevin thermostat, we show explicitly that indeed the effects of knot chirality become more evident for larger chain stiffness.

The behavior of the two knots is described in terms of their linear distance along the chain, and through the analysis of the system’s free energy (expressed in terms of this measure), we are able to identify three different regimes. In the first regime, the knots are separated along the polymer and the free energy is dominated by an entropic interaction between them. The second regime appears when the knots are close to each other but still not intertwined, and their interaction is dominated by the steric repulsion of the loops. The third and most intriguing regime emerges when the two knots are intertwined: in it, the free energy of the system depends on both the bending energy of the polymer and on the relative chirality of the knots.

These three regimes are rationalized by means of simple, mechanistic models based on the various contributions of different knotted configurations to the total entropy and/or elastic energy of the chain. In spite of their crudeness, these models illuminate the nature of chirality-dependent interactions between knots in stretched stiff polymers, and provide a solid starting point for further theoretical developments, as well as for the design of systems in vivo or in silico with specific, tailored physical properties.

## 2. Materials and Methods

### Model and Simulation Methodology

We model a single stiff linear polymer chain under external tension as a sequence of N=300 spherical beads attached to and stretched between two impenetrable planar hard walls, approximating the polystyrene beads used in a typical optical tweezer experiment [35]. The polymer excluded volume interaction is accounted for by a purely repulsive Weeks-Chandler-Anderson (WCA) potential between each pair of beads, while the connectivity of the chain is described by Finite Extension Non-linear Elastic (FENE) bonds [36,37]. The bending stiffness of the polymer chain enters through an elastic filament curvature deformation energy depending on the relative angle between two successive links along the chain.

The total potential energy of the system can therefore be expressed as a sum of four components:

(1) $$\begin{array}{c} U_{\mathrm{tot}}=U_{\mathrm{WCA}}+U_{\mathrm{FENE}}+U_{\mathrm{bending}}+U_{\mathrm{walls}} \end{array}$$

where the WCA potential is taken as:

(2) $$U_{\mathrm{WCA}}=\frac{1}{2}\sum_{(i,j),j\neq i}^{N}V\left(r_{i,j}\right)$$

with

(3) $$V\left(r\right)=\left\{\begin{array}{c} 4\epsilon \left[{\left(\frac{\sigma }{r}\right)}^{12}-{\left(\frac{\sigma }{r}\right)}^{6}+\frac{1}{4}\right]\;\mathrm{for}\;r\leq 2^{1/6}\sigma \\ 0\;\;\mathrm{otherwise} \end{array}\right..$$

The WCA interaction strength $\epsilon =1k_{B}T$ and the characteristic length scale *σ* are taken as the energy and length units, respectively. All other dimensional quantities are expressed in terms of reduced units defined through *ϵ*, *σ*, and the bead unit mass *m*. Time is measured in the MD time units $\tau_{\mathrm{MD}}=\sigma \sqrt{m/\epsilon }=1$. The FENE potential is given by:

(4) $$\begin{array}{c} U_{\mathrm{FENE}}=-\sum_{i=1}^{N-1}\frac{\kappa_{\mathrm{FENE}}}{2}{\left(\frac{R_{0}}{\sigma }\right)}^{2}\ln\left[1-{\left(\frac{|u_{i}|}{R_{0}}\right)}^{2}\right] \end{array}$$

where $u_{i}\equiv r_{i+1}-r_{i}$ is the connecting vector between bead i+1 and bead *i* (directed towards the former), and the modulus $|u_{i}|$ is thus the separation between the centers of beads *i* and i+1. The value of the maximum bond length $R_{0}=1.5\sigma$ and the FENE interaction strength $\kappa_{\mathrm{FENE}}=30\epsilon$ are the customary values pertinent to the Kremer–Grest model [36]. Finally, the elastic bending potential is taken in the form:

(5) $$U_{\mathrm{bending}}=\sum_{i=1}^{N-1}\kappa_{b}\left(1-\frac{u_{i}\cdot u_{i+1}}{|u_{i}\left|\right|u_{i+1}|}\right)$$

where $\kappa_{b}$ is the bending rigidity of the chain.

The full interaction potential of Equation (1) is employed in underdamped MD simulations in an implicit solvent. The simulation time step is $\Delta t=0.01\tau_{\mathrm{MD}}$, and the friction self-correlation time is $\tau_{\mathrm{frict}}=10^{3}\tau_{\mathrm{MD}}$. For each system under examination, we run 30 independent simulations, each covering $2\times 10^{7}\tau_{\mathrm{MD}}$, with an initial equilibration phase of length $2\times 10^{5}\tau_{\mathrm{MD}}$. The simulations were performed making use of the LAMMPS [38] MD package.

The confining hard walls and the effective impenetrability of the chain bonds—Provided by the combination of the FENE potential and the WCA interaction—Ensure that the topology of the system remains fixed to the one set by the initial configuration of two trefoil knots; thus, either $3_{1}^{+}3_{1}^{-}$ (+−) or $3_{1}^{+}3_{1}^{+}$ (++). The + and − superscripts indicate the handedness of the knots, right or left, according to the sum of the signed crossings in their minimal diagrams [39,40]. Since in our setup (++) and (−−) knots are related by a mirror transformation, their physical properties are equivalent, and we only simulate the former.

To study the dependence of the chirality effects on the bending energy, we simulate each of the two setups with different chirality at various values of the chain stiffness; namely, $\kappa_{b}=2.5\;\mathrm{to}\;20.0\;k_{B}T$. In all setups, the termini of the chain are kept fixed at a distance of L=205σ, corresponding to stretching forces of about 1–7 pN at T=300 K, depending on the bending rigidity of the chain, and applied at both termini.

**Figure 1:**
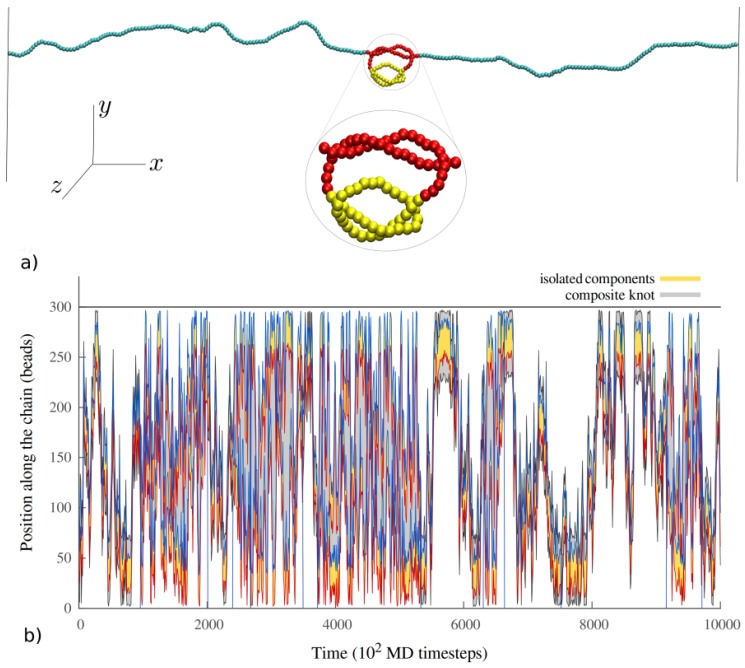
(**a**) An example of a configuration for the (++) system, with both knots intertwined. In this configuration, only one prime component(marked in yellow) is identified as “isolated” by our knot identification scheme based on the Minimally Interfering Closure [41]; (**b**) A small portion of an MD trajectory for the (++) system. Yellow shaded regions indicate the portions of the chain occupied by the isolated prime knots, and red and blue lines indicate their first and last bead, respectively. The portion of the chain taken up by the whole composite knot is reported as a gray shaded area. When the knots are intertwined, our algorithm identifies only one isolated knot, which can be seen in the trajectory as a single yellow region surrounded by gray boundaries. Note that the intertwined composite knot can still travel from one side of the chain to the other.

In order to study the statistical properties of the knots on the polymer chain, we need to identify which portions of the chain host the knots. The identification of the smallest knotted portion of the chain (that is, the segment that we define as the knot) is enabled by the usage of the Minimally Interfering Closure [41] to circularize open subsections of the chain into auxiliary arcs, whose topology is then established by means of the Alexander determinants in −1 and −2. It is worth note that the inability of the Alexander polynomial to distinguish different chiralities does not affect our results, since we are interested only in the distance between the two trefoil knots. We define a knotted portion of the chain as the shortest segment featuring a specific knotted topology. With some abuse of language, we will refer to such portions as “knots” in what follows. By applying this procedure to composite knots, we are able to identify both the chain portions hosting the whole composite knot, as well as those hosting its “isolated” prime components. Following Ref. [31], we consider a prime component to be isolated when it can be excised, and its ends joined, without at the same time untying the second knot (as depicted in Figure 1).

## 3. Results

We investigate the behavior of two trefoil knots on semiflexible chains of different bending rigidity, stretched between two impenetrable walls kept at fixed distance. The investigated bending rigidities span the range from $\kappa_{b}=2.5$ to $\kappa_{b}=20\;k_{B}T$, corresponding to different stretching forces, all in the strong stretching regime (see Methods). For each value of $\kappa_{b}$, we ran 30 independent simulations of about $10^{7}\tau_{\mathrm{MD}}$ steps each. We sampled the system by storing the whole polymer configuration every $100\tau_{\mathrm{MD}}$. A typical portion of an MD trajectory for $\kappa_{b}=20\;k_{B}T$ is reported in Figure 1b.

The knot localization scheme described in the Methods section allows us to easily distinguish configurations in which the knots are intertwined; i.e., when one knot is inside the other, from configurations in which they are separated along the chain. These states can be distinguished from the number of isolated components identified by our knot localization algorithm: two, when the knots are separated, and one—the nested knot—when the knots are intertwined. At $\kappa_{b}=2.5\;k_{B}T$, we do not observe any crossing event in which the two knots would become intertwined. Therefore, this value of $\kappa_{b}$ is considered in the following only in relation to the properties of the knots when they are separated along the polymer chain. At $\kappa_{b}=5\;k_{B}T$ we observe ≈30 crossing events in which the two knots get either intertwined or separated. The number greatly increases with $\kappa_{b}$, to reach ≈2500 events at $\kappa_{b}=20\;k_{B}T$, for a simulation of about ∼$5.6\times 10^{8}\tau_{\mathrm{MD}}$.

### 3.1. Knots Sizes

**Figure 2:**
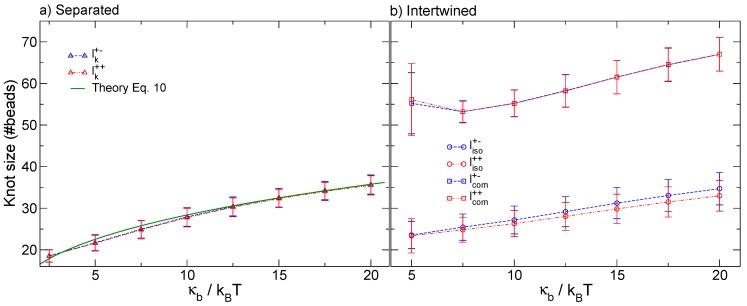
(**a**) Size of separated trefoil knots, plotted as a function of $\kappa_{b}$; (**b**) Size of a nested trefoil knot (circles) as well as of the composite knot (squares) when the two trefoils are intertwined, plotted as a function of $\kappa_{b}$.

The first observable on which we direct our attention is the size of the knots, measured as the number of beads included in the entanglements—Both in their separated and intertwined states—In dependence of the bending stiffness $\kappa_{b}$. From Figure 2a, we observe that the size of separated knots grows sublinearly with increasing bending rigidity. This is consistent with a simple minimization of the bending energy stored in the loop and braids of the knot, compounded with the effective chain shortening as some of its length is used up by the knots [42,43]. More interestingly, it appears that the size of the composite, intertwined knot, (shown in Figure 2b) depends on the bending rigidity in a non-linear, possibly also non-monotonic fashion, with the knot size deviating substantially from a linear dependence for small values of $\kappa_{b}$. This may be related with recent observations by Poier [22] and Caraglio [26], and points to the importance of the entropic effects that become more important as the bending rigidity is diminished.

Neither the size of the separated knots nor that of the whole intertwined knots manifest a dependence on the relative chirality. This is not the case for the size of the nested knot, as can be appreciated from Figure 2b. Here, in contrast with the whole intertwined knot, we have a substantially linear growth of the nested knot size with $\kappa_{b}$, and the sizes of the nested knots for the different chirality—Initially having the same value, $\kappa_{b}=5\;k_{B}T$—Increase with different rates. Specifically, the nested knot in the setup where both prime components have the same chirality shows a smaller size with respect to the (+−) case. Albeit small, this discrepancy points to distinguishably different organizations of the intertwined knots depending on their relative chirality, and we substantiate this expectation in the following.

### 3.2. Knots Free Energy

**Figure 3:**
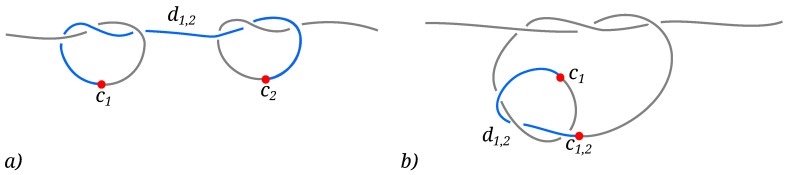
Schematics of the collective order parameter |D| measuring the linear distance between two prime knots. (**a**) When both prime components are isolated , the order parameter is given by $\left|D|=\right|d_{1,2}\left|=\right|c_{1}-c_{2}|$, where $c_{i}=\left(e_{i}+s_{i}\right)/2$ is the center of knot *i* on the chain. Here $e_{i}$ and $s_{i}$ stand for the last and the first bead of the *i*-th isolated prime knot; (**b**) When the two knots are intertwined, the center of the hosting knot is taken to coincide with the center of the composite knot $c_{1,2}$.

We proceed to study how the free energy of the (+−) and (++) systems depends on the different arrangements of the knots along the chain. To do so, we investigate the free energy as a function of a collective order parameter |D|, defined as the absolute linear distance between the centers of the two knots [32], see Figure 3. Specifically, we identify the starting and ending beads $s_{i}$ and $e_{i}$ of both knots on the chain, where the index *i* pertains to the knot; the center of knot *i* along the chain is then at $c_{i}=\left(s_{i}+e_{i}\right)/2$. When only one trefoil can be identified by the Minimally Interfering Closure (see Methods section), the knots are intertwined and we can take the starting and ending beads of the non-isolated knot to correspond to those of the whole composite knot. Consequently, the collective order parameter can be introduced as the absolute linear distance between the centers of the two knots, defined as:

(6) $$\left|D\right|=\left\{\begin{array}{cc} |c_{1}-c_{2}| & \left(\mathrm{separated}\mathrm{knots}\right)\\ |c_{1}-c_{1,2}| & \left(\mathrm{intertwined}\mathrm{knots}\right) \end{array}\right.$$

**Figure 4:**
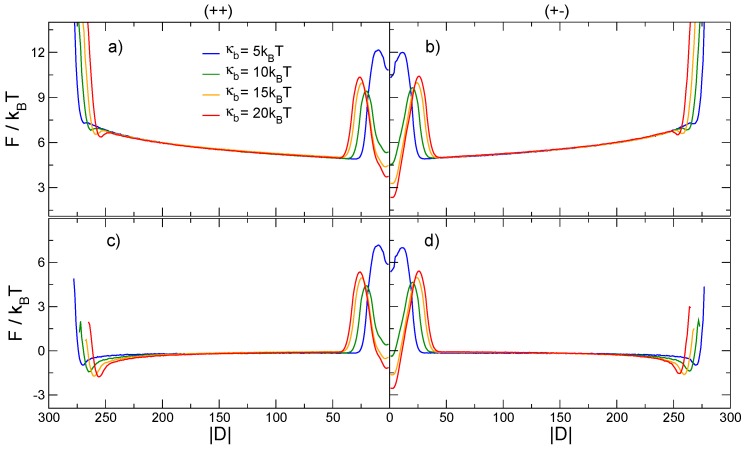
Free energy *F* as a function of the collective order parameter |D|, i.e., the absolute linear distance between the centers of the two knots. F(|D|) for different values of the chain bending rigidity $\kappa_{b}$ is reported for (**a**) two trefoil knots with the same relative chirality and (**b**) two trefoil knots with opposite relative chiralities. In (**c**,**d**) we show the same free energies, but with subtracted entropic contribution −TS(|D|), as defined in Equation (8).

Starting from the definition of |D|, we evaluated the free energy $F\left(|D|\right)=-k_{B}T\log\left(p(|D|)\right)$, where p(|D|) is the probability of finding the knots at distance |D|. The free energy profiles F(|D|) for the (++) and (+−) systemsat various values of the polymer bending rigidity are reported in Figure 4. Consistent with previous results [32,34],we observe that the intertwined state with |D|=0 becomes a global minimum only when the bending rigidity $\kappa_{b}$ reaches large enough values. We observe that such values differ for knots with the same or different chiralities. In addition, due to the high stretching of the polymer chain, the intertwined state of either chirality configuration becomes a global minimum only when $\kappa_{b}>10\;k_{B}T$, a higher value than the one observed in Ref. [32] for a more complex setup ($3_{1}4_{1}$), but at lower stretching forces.

The plots of Figure 4 also indicate that the (+−) system always has a deeper minimum than the (++) system, while the rest of the curves remain effectively universal and independent of the bending rigidity, with a slow growth of the free energy with increasing |D|. This universal behavior is eventually followed by a small minimum for very high separations of the two knots, corresponding to states in which each knot is localized in the vicinity of a confining wall. Increasing |D| even further, the free energy monotonically increases up to a point at which |D| is close to the maximum possible separation between the knots. The different behaviors of F(|D|) for large values of |D| are due to finite size effects. In particular, the minima and barriers at |D| close to N=300 are caused by the presence of the impenetrable walls, while the universal slow growth of F(|D|) originates from the finite size of the chain itself and can arguably also be observed on unstretched rings [31]. Similar results(excluding the minima and the barriers due to the presence of the walls) were obtained by excluding all configurations in which one knot was closer to the wall than $\langle l_{k}\rangle$ (data not shown).

The universal bending rigidity-independent part of the free energy as a function of the order parameter |D|—In the range of values between the intertwined knot state and the knot localization at the boundaries of the chain—Can be understood with simple scaling arguments. Assuming that the length of the a trefoil knot ($l_{k}$) does not fluctuate significantly from its average value $\langle l_{k}\rangle$ when the knots are separated, we can map the polymer chain onto a linear string of *N* beads, along which two chosen segments of length $s=\langle l_{k}\rangle$ (representing the knots) can slide freely. From the definition of |D|, we can see that the two knots of equal length separated by |D| beads take up a portion of the chain of size $\alpha =|D|+\langle l_{k}\rangle$. An explicit counting of the available microstates shows that there are $N-\langle l_{k}\rangle +1-|D|$ configurations in which the segments are separated by a distance |D| between their centers, without any double counting, since the knots are indistinguishable when separate. The fraction of microstates $\omega (|D|;N,\langle l_{k}\rangle )$ can then be written as:

(7) $$\begin{array}{ccc} \omega (|D|;N,\langle l_{k}\rangle ) & = & \frac{\left(N-\langle l_{k}\rangle +1-|D|\right)}{\sum_{|D|=0}^{N-\langle l_{k}\rangle +1}(N-\langle l_{k}\rangle +1-\left|D|\right)}\\ & = & \frac{2(N-\langle l_{k}\rangle +1-|D|)}{\left(N-\langle l_{k}\rangle +2\right)\left(N-\langle l_{k}\rangle +1\right)} \end{array}$$

and the entropy as a function of |D| reads:

(8) $$S(|D|;N,\langle l_{k}\rangle )=k_{B}\ln\omega (|D|;N,\langle l_{k}\rangle ).$$

Subtracting this purely entropic contribution from the free energies in Figure 4a,b and making use of the values of $\langle l_{k}\left(\kappa_{b}\right)\rangle$ reported in Figure 2, we obtain a completely flattened-out free energy for |D| between the intertwined knot state and the knot localization at the boundaries of the system. This simple transformation of the free energy accentuates those non-universal features that depend on the bending rigidity, and consequently on the interactions between the two knots or between the knots and the confining walls; see Figure 4c,d.

**Figure 5:**
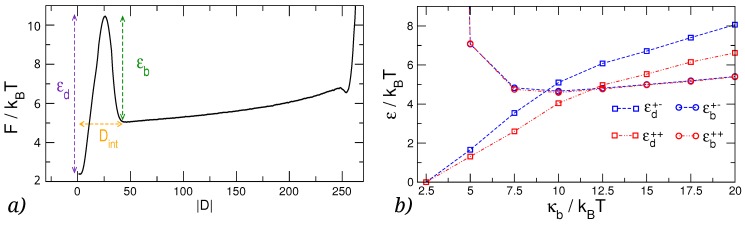
(**a**) Graphical representation of three quantities discussed in the text: the height of the barrier, $\epsilon_{b}$, the depth of the minimum, $\epsilon_{d}$, and the interaction distance, $D_{\mathrm{int}}$; (**b**) Dependence of the height of the barrier and the depth of the free energy minimum on the polymer bending rigidity, $\kappa_{b}$.

From the plots in Figure 4a,b, we extract the ΔF between the minimum and the barrier ($\epsilon_{d}$), the height of the barrier with respect to the entropic plateau ($\epsilon_{b}$), and the distance $D_{\mathrm{int}}$ at which the knots begin to interact. The latter is defined as the value of |D| at which the F(|D|) starts to increase when |D| is reduced below |D|=N/2, see Figure 5a. The results for ΔF, reported in Figure 5b, show that the height of the barrier ($\epsilon_{b}$), does not depend on the relative chirality of the two knots, but displays an interesting dependence on $\kappa_{b}$. In fact, $\epsilon_{b}$ appears to have a very broad minimum for $\kappa_{b}∼10\;k_{B}T$. On the other hand, we observe that the depth of the minimum ($\epsilon_{d}$), increases monotonically with $\kappa_{b}$, and that the separation between the $\epsilon_{d}$ curves for (++) and (+−) knots increases as well, confirming that the emergence of the chiral effect is finally triggered by the bending rigidity of the chain.

**Figure 6:**
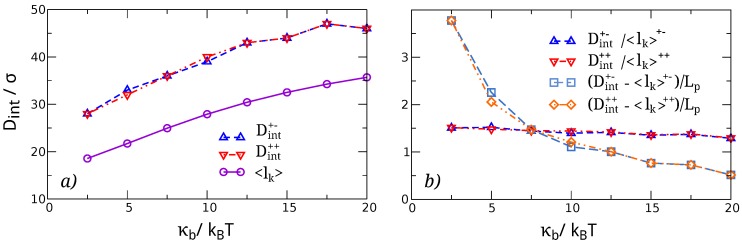
(**a**) Interaction distances of separated knots as a function of $\kappa_{b}$. For comparison, we also report the knot size averaged between the (++) and (+−) systems, $\langle l_{k}\rangle$. Note that the interaction distance is always larger than $\langle l_{k}\rangle$; (**b**) Two different rescaling of $D_{\mathrm{int}}$: $D_{\mathrm{int}}/\langle l_{k}\rangle$ and $\left(D_{\mathrm{int}}-\langle l_{k}\rangle \right)/L_{p}$, with $L_{p}$ being the persistence length of the chain.

In light of these results, one is naturally led to wonder if the bending energy of the chain could be implicated in mediating an effective interaction between the knots when they are separated as well. To investigate this aspect, we identified the interaction distance between two well-separated knots. From the data reported in Figure 6a, we can see that indeed the knots begin to interact at distances $|D|>\langle l_{k}\rangle$, the characteristic distance at which their ends along the chain coincide. In order to see if this interaction is dictated by the curvature of the chain segment connecting the two knots and its fluctuations, or by the steric interaction between the two knot loops, we rescale $D_{\mathrm{int}}$ by $\langle l_{k}\left(\kappa_{b}\right)\rangle$. The results reported in Figure 6b show that $D_{\mathrm{int}}$ increases slightly slower than $\langle l_{k}\rangle$ with *k*. Since $\langle l_{k}\rangle$ grows sublinearly with the persistence length $L_{p}∼\sigma \kappa_{b}/k_{B}T$, one can immediately see that $\frac{D_{\mathrm{int}}}{L_{p}}$ decreases faster than $\frac{D_{\mathrm{int}}}{\langle l_{k}\rangle }$ with $\kappa_{b}$. The same goes for the length of the segment connecting the two knots, $D_{\mathrm{int}}-\langle l_{k}\rangle$, as can be seen from Figure 6b. From this analysis, we conclude that the interaction between two simple separate knots on a stretched semiflexible polymer of bending rigidity up to $L_{p}∼20\sigma$ is dictated primarily by the steric hindrance of their loops.

### 3.3. Relative Orientation of the Knots

An important descriptor of the organization of the knots is their relative orientation when they are separated, as well as when they are nested. We thus introduce the knot orientation director $U_{k}$, defined as the sum of the vector products of consecutive bond vectors; the sum is extended to all bond vectors contained in the knot:

(9) $$U_{k}=\frac{\sum_{j\in k}u_{j}\times u_{j+1}}{|\sum_{j}u_{j}\times u_{j+1}|}.$$

**Figure 7:**
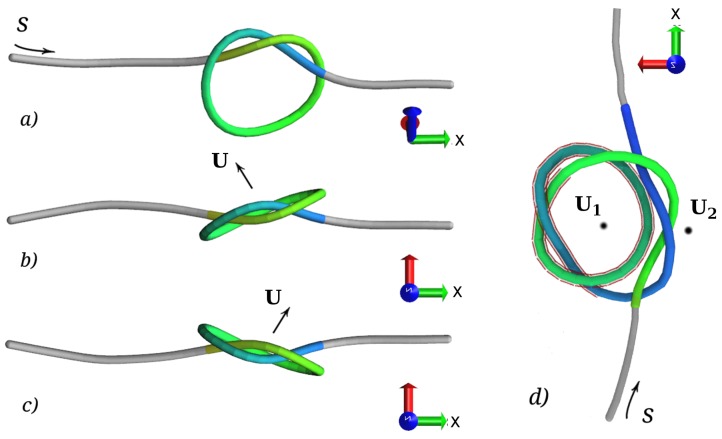
Left-handed $3_{1}$ knot configuration obtained by minimizing the energy viewed from the side (**a**) and from the top (**b**); (**c**) Right-handed $3_{1}$ knot viewed from above. *X* indicates the pulling direction. In (**a**), the direction of the arc length *s* is indicated by an arrow, and in all panels by the coloring of the knots, from green to blue for increasing *s*. In panels (**b**,**c**), we reported the direction of the vector $U_{k}$ defined in Equation (9). Note that $U_{k}\cdot x$ changes sign with the handedness of the knot; (**d**) A (+−) intertwined composite knot, with the nested knot highlighted by a red shading. Note that when the loop of the nested knot lies inside the loop of the outer knot, both orientation directors point out of the page. On the other hand, when the loop of the two knots form an eight, the two directors point in opposite directions.

The direction of $U_{k}$ is defined by the arc length along the knot *k*, according to the right hand rule. In configurations obtained by minimizing the energy of the knot, $U_{k}$ identifies the normal to the plane passing trough the knot loop, as depicted in Figure 7. Note that knots of different handedness have opposite projections of $U_{k}$ along the pulling direction *X*.

Using the knot orientation director, we introduce two order parameters, which are based on idealizing the knot loops as rigid discs. The first such parameter, *θ*, captures the (instantaneous) angle between two knot loops, and is defined as:

(10) $$\begin{array}{c} \theta =\arccos(U_{1}\cdot U_{2}). \end{array}$$

Since *θ* measures only the aperture of the cone identified by $U_{1}$ and $U_{2}$, we consider another parameter to fully capture the relative orientation of the two knots, $\theta_{⊥}$, which captures the relative rotation of the knot loops with respect to the stretching axis *X*:

(11) $$\begin{array}{c} \theta_{⊥}=arctan2\left(sgn\;|V_{1}\times V_{2}|,V_{1}\cdot V_{2}\right) \end{array}$$

with:

$$\begin{array}{c} V_{i}\equiv \frac{\left(0,U_{i}^{y},U_{i}^{z}\right)}{\sqrt{{\left(U_{i}^{y}\right)}^{2}+{\left(U_{i}^{z}\right)}^{2}}}\\ sgn=sign({\left[U_{1}\times U_{2}\right]}^{x}) \end{array}$$

In words, $\theta_{⊥}$ measures the angle between the projections of the U vectors on the YZ plane (that is, the plane perpendicular to the stretching direction). These projections constitute the normalized two-dimensional vectors V. The sign of the angle, which can be determined by means of the arctan2 function (analogous to the two-argument FORTRAN routine ATAN2 for the inverse tangent), is determined by the *X* component of the vector product between the full U vectors: if the resulting vector points in the same direction as *X*, the sign is positive.

When the two knots are intertwined, $U_{1}=U_{\mathrm{nested}}$ is defined for the bonds of the nested knot, while $U_{2}$ is defined for the bonds pertaining to the region of the composite knot that is complementary to the nested knot. This allows us to use the same orientational parameters both when the knots are separated, and when they are nested.

**Figure 8:**
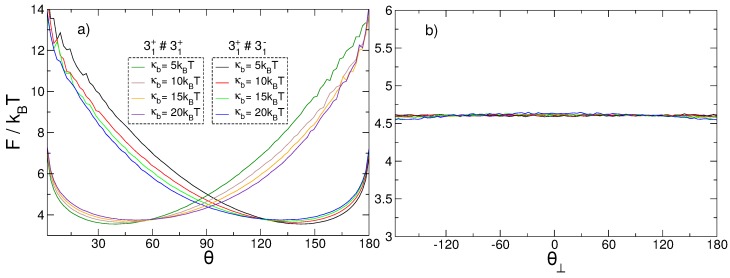
Free energy of two separated trefoil knots projected along (**a**) *θ* and (**b**) $\theta_{⊥}$ for four different values of polymer bending rigidity.

We first study the relative orientation of the knots when they are separated. From the data in Figure 8a, we notice that the projection of the free energy along *θ* presents broad minima for angles close to π/4 for the (++) system and angles close to 3π/4 for the (+−) system. The minimum energy angles $\theta^{*}$ for the two systems are symmetric with respect to π/2; i.e., $\theta_{\left(++\right)}^{*}=\pi -\theta_{\left(+-\right)}^{*}$. On the other hand, it is clear from Figure 8b that there is no preferred relative orientation of the two loops on the YZ plane. The minima of F(θ) thus simply capture the different alignment of the braids of the two knots, which depends on the chirality of the knots, as shown in Figure 7.

**Figure 9:**
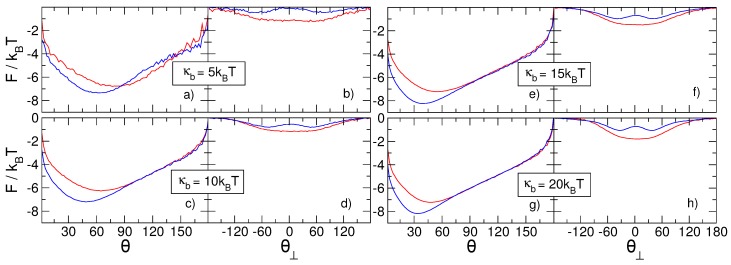
Free energy of two intertwined trefoil knots with the same handedness (red curves) and opposite handedness (blue curves) as a function of *θ* (**a**,**c**,**e**,**g**) and $\theta^{⊥}$ (**b**,**d**,**f**,**h**), for four different values of polymer bending rigidity: $\kappa_{b}=5$, $\kappa_{b}=10$, $\kappa_{b}=15$, and $\kappa_{b}=20k_{B}T$. The free energies have been shifted so that their maximum values correspond to zero.

When the knots are intertwined, we expect that their arrangement will be sensitive to the their relative chirality. Since the difference between the depth of the minima of the (++) and (+−) systems increases with increasing bending rigidity, we further expect the arrangement of the intertwined knots in the two systems to become more distinguishable when the polymer stiffness is increased. As shown in Figure 9, this is indeed the case. Interestingly, we observe that both systems present a free energy minimum for a value of *θ* between 30° and 45°, suggesting that their loops tend to be one inside the other; this minimum is more pronounced and corresponds to smaller angles for the (+−) system, suggestive of more planar configurations. Furthermore, the minimum energy angle $\theta^{*}$ decreases with increasing bending energy, meaning that the aperture of the cone between the two vectors decreases with increasing polymer stiffness. The knots therefore get more aligned with increasing bending energy, and for opposite relative chiralities.

Investigating the behavior of $\theta_{⊥}$, we note that while the (++) system presents a minimum in $\theta_{⊥}=0$, the (+−) system presents two minima for $\theta_{⊥}∼\pm 30{}^{\circ}$. The minima are shallow in both systems, and therefore all values of $\theta_{⊥}$ are easily explored, although the barriers seem to be lower in the (+−) system. The presence of two minima in the (+−) system can be understood considering that for it there can be two distinct arrangements of the intertwined knots: one in which the $3_{1}^{+}$ is nested inside the $3_{1}^{-}$ knot, and the other in which the opposite happens. For every configuration with the $3_{1}^{+}$ nested inside the $3_{1}^{-}$, there is a corresponding specular configuration in which the handedness of the two knots is reversed and the $3_{1}^{-}$ is nested inside the $3_{1}^{+}$. These specular configurations have the same Boltzmann weight. Given our definition of the sign of $\theta_{⊥}$, these two arrangements (being specular) will correspond to opposite values of $\theta_{⊥}$. The values of $\theta_{⊥}$ in which two minima are located are almost exactly coincident to those for which the free energy of the (+−) system as a function of *θ* has its minima (see Figure 9a,c,e,g). This is suggestive of the fact that the U directors lie mainly in the YZ plane, so that their projections form the same angle as the three-dimensional vectors themselves. On the contrary, the broad, single minimum in $\theta_{⊥}=0{}^{\circ}$ of the free energy of the (++) system is due to a more skewed arrangement of the nested knot, whose director—Together with that of the larger knot—Identifies a plane roughly perpendicular to the YZ plane; the projections of the two directors thus form a quite small angle—Zero on average—In spite of a wider angle θ∼45° in the three dimensional space. This arrangement is consistent with the two intertwined prime components of the (++) system to be less coplanar.

## 4. Discussion

The detailed analysis of the free energy of semiflexible chains under strong external tension as a function of various collective order parameters has led us to identify several different regimes:

- When the knots along the chain are clearly separated and sufficiently far from the hard walls, the free energy is dominated by an *entropic interaction* between the knots, dependent on the absolute linear distance between them as well as the length of the knots.
- As the knots get closer to one another, but can still be considered as two simple separate knots, a repulsive interaction starts to dominate the free energy, stemming primarily from the steric hindrance of the proximal loops of the knots.
- Finally, as the knots become intertwined at yet smaller effective separations, the absolute magnitude of the free energy—And consequently, the stability of the knotted chain configuration—Is dependent both on the bending stiffness of the polymer chain as well as on the *relative chirality* of the two knots.

In what follows, we will provide simple, mechanistic reasons for this behavior based on considerations of the various contributions of different knotted configurations to the total elastic energy of the chain.

### 4.1. Elastic Energy Model for the Size of Two Separate, Non-Interacting Knots

We start our discussion by observing that the behaviour of two separated knots along a polymer, stretched by high enough imposed forces to localize the knot, is essentially dictated by the knot length, as indicated both by Equation (8) and by the fact that the interaction distance scales with the knot length, as reported in Figure 6. It is therefore of interest to derive a simple expression for the expected knot length as a function of the bending rigidity of the chain.

The bending rigidity of a knot can be computed by modeling it as a loop of radius *R*, and taking into account that its length (as provided by the location algorithm) is not equal to 2πR, but also includes the braided region. We approximate the extent of the braided region with a segment of length ∼2*R*, and assume it has the same curvature as the rest of the knot. These approximations lead to a knot length L=(2π+2)R, and the radius (expressed as a function of the length) as R=L/(2π+2). The bending energy then follows as:

(12) $$\begin{array}{ccc} E_{b} & = & \int_{L}dl\;\frac{1}{2}\kappa_{b}{\left(\frac{1}{R}\right)}^{2}=\frac{2{\left(\pi +1\right)}^{2}\sigma \kappa_{b}}{n} \end{array}$$

where we used L=σn, with *n* number of beads of the knot and *σ* the bond length.

The (effective) free energy of the knotted chain as a function of the knot length is given by the sum of the bending energy contribution of each knot, plus the contribution of the whole chain due to external tension. For the latter, we assume that a linear force acts on the knot as a function of its size, thus leading to a quadratic free energy of the form [42]:

(13) $$\begin{array}{ccc} E_{t} & = & \frac{1}{2}Qn^{2}, \end{array}$$

where *Q* is an as-yet unspecified constant. The total free energy of two independent and equivalent knots is then given by:

(14) $$\begin{array}{ccc} E_{\mathrm{tot}} & = & \sum_{i=1,2}\left(\frac{2{\left(\pi +1\right)}^{2}\sigma \kappa_{b}}{n_{i}}+\frac{1}{2}Qn_{i}^{2}\right). \end{array}$$

To compute the average size of the knots, we investigate the minimum of $E_{\mathrm{tot}}$ with respect to $n_{1},n_{2}$. We get straightforwardly that the above free energy is minimized by:

(15) $$\begin{array}{c} n_{1,2}^{☆}={\left(\frac{2{\left(\pi +1\right)}^{2}\sigma \kappa_{b}}{Q}\right)}^{1/3}. \end{array}$$

The value of *Q* can then be obtained by fitting the numeric knot size; e.g., taking the value of n∼36 for $\kappa_{b}=20$, see Figure 2a. This gives us Q=0.015. The resulting function, reported in Figure 2a, is in very good agreement with the data obtained in the simulations.

### 4.2. Elastic Energy Model for Chirality Effects in Knot–Knot Interaction

Having analytically characterized the behaviour of two separated knots on a semiflexible chain under external tension, we now proceed to discuss the underpinnings of the dependence of the stability of the intertwined state on the relative chirality of the two knots. While a general elucidation and the corresponding analytical formulation of this effect will be hard to come by—Being dependent on the complex details of the polymer configuration—We nevertheless offer a simple insight which to some extent clarifies the ultimate mechanical reason giving rise to this phenomenon, without delving too deeply into all the pertinent details.

**Figure 10:**
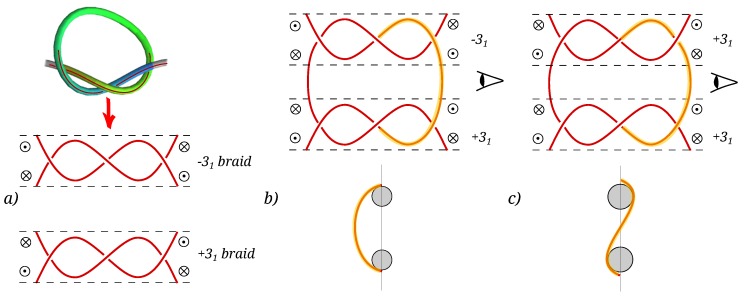
(**a**) A trefoil knot can be decomposed into two loops and a braid. The two enantiomers are defined by the braid. The circles indicate the orthogonal components of the bending force vector. Following the usual conventions, crossed circles indicate chain directors going into the page, while dotted circles indicate chain directors coming out of the page; (**b**,**c**): Composing the intertwined knots of equal and/or different chiralities. In an intertwined (+−) knot (**b**), the director joins the junction between the braid and the loop in the same sense, while in an intertwined (++) knot (**c**), it points in opposite sense at the two junctions; (**b**,**c**) bottom: Schematic representation of the axial projection (in the direction of the braid axes, assumed to coincide) for the (+−) and (++) composite knots, respectively.

We start by observing that a torus knot braid (the portion of the knot containing its essential crossings)can be modeled as a helix, as can be seen from Figure 10. For simplicity, we envision the two trefoil knots as having their braids lying in the same plane (i.e., the axes of their helices are coplanar). We now concentrate on the portion of the chain connecting the two braids, colored in the upper part of panels (b) and (c) of Figure 10. Rotating the axes of the braids in such a way that they are directed into the page, we obtain the schematics of the lower part of panels (b) and (c). Obviously, the trajectory of the chain segment connecting the two is quite different for the case of opposite and equal chiralities. In order for the segment of the chain to seamlessly connect the two braids, its spatial configuration must be fundamentally different, as indicated in the bottom parts of panels (b) and (c).

The reason why two types of composite knots can have different elastic energies can then be gleaned from a highly idealized configuration, where the composite knot is presented by loops and braids (Figure 10). Preserving the chirality distribution in the composite knot, one can discern that the elastic energy of a simplified (+−) configuration has to be smaller than for the (++) configuration. Concentrating exclusively on the segment connecting the two braids in the composite knot, we can write its bending energy as:

(16) $$E_{b}=\int_{L}dl\;\frac{1}{2}\kappa_{b}{\left(\frac{1}{R}\right)}^{2}=\int_{L}dl\;\frac{1}{2}\kappa_{b}{\left(\frac{d\psi (l)}{dl}\right)}^{2},$$

where ψ(l) is now the angle between the vertical axis and a position on the connecting segment at *l*. The minimization of this bending energy (leading to $d^{2}\psi \left(l\right)/dl^{2}=0$) should be accompanied by the appropriate boundary conditions and symmetry of the solution ψ(l). This problem also bears some distant similarity to the wrapping transition and wrapping-mediated interactions for discrete binding along an elastic filament [44], where the role of the wrapping adsorbands would be played by the constituent braids of the knot.

Clearly, the (++) configuration corresponds to a node of a curvature, while the (+−) configuration does not. In general, nodes make the energy higher, and we therefore conclude that the (++) configuration would have a higher bending energy. One must anyway keep in mind that this argument is only approximate and would need to be refined, since we are not allowing the braids to relax in order to minimize the bending energy. Admittedly, then, the above argument is very simplistic, but sufficiently robust to explain the observed knot interaction energies obtained with a full MD tracing of the partition function of the knots.

## 5. Conclusions

By performing detailed underdamped MD simulations of a constrained semiflexible chain under strong external tension, we investigated its free energy as a function of a properly-defined collective order parameter, |D|, quantifying the absolute linear distance between the centers of two knots along the chain. We discovered several regimes in this dependence, characterized by the relative importance of entropy, bending energy, and end constraints. In each of these regimes, the free energy depends in a characteristic way on |D|.

The first regime is characterized by knots being clearly separated along the chain; i.e., no intertwining, with the free energy being dominated by a non-specific, entropic interaction between them as well as by the end constraints when the knots are close to the confining walls. When the separation between knots diminishes, a repulsive interaction emerges due to the steric hindrance between close loops of the knots. As the knots start to intertwine at smaller effective separations, the free energy is dominated by the bending energy, dependent both on the bending stiffness of the polymer chain as well as on the relative chirality of the two knots.

Emergence of chirality as the defining factor for the stability of intertwined knots is the most important corollary of our work. In this sense, the knots behave in a fundamentally different way from sliplinks [45], obviously bearing another degree of freedom that affects their interaction in a fundamental manner.

We analyzed the intertwined state by introducing two further order parameters, *θ* and $\theta_{⊥}$, which capture the relative orientation between the two knots. Mapping the free energy of the system onto them, we observed that two intertwined trefoils tend to have their loops one inside the other, and that this tendency increases with increasing bending energy. Furthermore, two trefoil knots of opposite chirality tend to be in a more planar configuration than two trefoils having the same chirality.

Finally, we identified the mechanistic basis for the chiral effect in the intertwined state by observing that the braids of two torus knots of opposite chirality have different alignment with respect to the tensioned rope on which they are tied. Because of this, two knots of opposite chirality can form an intertwined state in which their braids are aligned without introducing any additional bending in the chain, since they are the specular image of one another. The same cannot happen for two knots of the same chirality, which can only be connected with their braids lying on the same plane by introducing a node in the curvature of the segments connecting the braids, thus increasing the bending energy.
